# Anti-oxidant and neuro-modulatory effects of bioactive *Byttneria pilosa* leaf extract in swiss albino mice using behavioral models

**DOI:** 10.3389/fchem.2024.1341308

**Published:** 2024-02-08

**Authors:** Mifta Ahmed Jyoti, Md. Shahin Shah, Mohammad Najim Uddin, Mohammed Kamrul Hossain, Aixia Han, Peiwu Geng, Mohammad Nazmul Islam, Abdullah Al Mamun

**Affiliations:** ^1^ Department of Pharmacy, International Islamic University Chittagong, Chittagong, Bangladesh; ^2^ Department of Pharmacy, Faculty of Life and Earth Science, Jagannath University, Dhaka,Bangladesh; ^3^ Department of Pharmacy, Faculty of Biological Sciences, University of Chittagong, Chittagong, Bangladesh; ^4^ Central Laboratory of The Sixth Affiliated Hospital of Wenzhou Medical University, Lishui People’s Hospital, Lishui, Zhejiang, China

**Keywords:** *Byttneria pilosa*, MEBP, antioxidant, polyphenols, anxiolytic, antidepressant, anti-diabetic

## Abstract

*Byttneria pilosa*, a flowering plant from the Malvaceae family traditionally used to treat ailments such as boils and scabies, is here investigated for its potential health benefits. The study focused on evaluating its antioxidant and antidiabetic properties *in vitro*, as well as the *in vivo* anxiolytic and antidepressant activities of the methanol extract of *B. pilosa* leaf (MEBP). The study employed various assays to evaluate antioxidant activity, including 2, 2-diphenyl-1-picrylhydrazyl (DPPH) radical scavenging, reducing power capacity, and quantification of the total phenolic and flavonoid contents of MEBP. Additionally, anxiolytic and antidepressant activities were evaluated through four tests: elevated plus-maze test (EPMT), light–dark box test (LDBT), forced swimming test (FST), and tail suspension test (TST). Antidiabetic effect was determined using α-amylase inhibition assay. Docking analysis was performed using BIOVIA and Schrödinger Maestro (v11.1), and the absorption, distribution, metabolism, and excretion/toxicity (ADME/T) properties of bioactive substances were investigated using a web-based technique. MEBP exhibited moderate antioxidant activity in DPPH radical scavenging and reducing power capacity assays, with a dose-dependent response. The total phenolic and flavonoid contents measured were 70 ± 1.53 mg and 22.33 ± 1.20 mg, respectively. MEBP demonstrated significant effects in α-amylase inhibition comparable to acarbose. In behavioral tests, MEBP dose-dependently altered time spent in open arms/light box and closed arms/dark box, indicating anxiolytic effects. Moreover, MEBP significantly reduced immobility duration in FST and TST, suggesting antidepressant properties. Molecular docking analysis revealed favorable interactions between beta-sitosterol and specific targets, suggesting the potential mediation of anxiolytic and antidiabetic effects. Overall, MEBP exhibits notable anxiolytic and antidepressant properties, along with moderate antioxidant and antidiabetic activities.

## Graphical Abstract

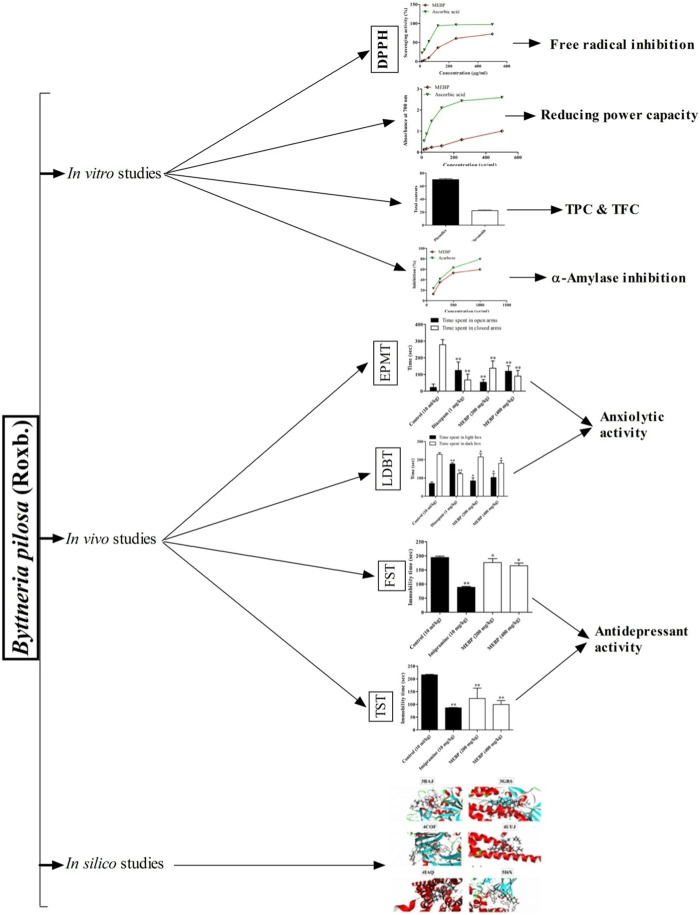

## 1 Introduction

Natural products and nutraceuticals, given their significantly elevated safety margin and cost-effectiveness, are attracting increasing attention for their potential as both preventive and therapeutic interventions in disease management. The spectrum of mental disorders, encompassing anxiety, stress, depression, and psychosis, poses significant challenges that adversely impact overall quality of life (Sarris et al., 2015). Free radicals inherently unstable molecules produced by the body in response to internal and external stresses pose a threat to cellular integrity. The damaging effects of free radicals on cells can be mitigated or prevented by antioxidants (Gutowski and Kowalczyk, 2013). Often referred to as free-radical scavengers, antioxidants are crucial in reducing the likelihood of inflammation and other health problems associated with free radicals. These compounds can be sourced both synthetically and naturally. Numerous studies suggest that plant-based diets are particularly rich in antioxidants, underscoring the importance of a diet abundant in phytonutrients derived from plants for combating oxidative stress and promoting overall health (Liu, 2004). In recent years, medicinal plants have been widely studied for their numerous effects, especially antioxidant activity. It is firmly believed that increasing the intake of food enriched in natural antioxidants lowers the risk of degenerative diseases, cardiovascular diseases, and cancer (Do et al., 2014; Rahman et al., 2020). Commercial antioxidants prepared in laboratories, such as propyl gallate, butylated hydroxyanisole, butylated hydroxytoluene, and tertiary hydroxyquinone, are extensively used in industry to control lipid oxidation (Banu et al., 2020; Emon et al., 2020). Due to their potential health risks and toxicity, the use of these synthetic antioxidants has been questioned (Wong et al., 2006). The major cause of most diseases is excessive ROS production (Uddin et al., 2020). Hence, researchers have focused on antioxidants from natural resources, and they continue to search for replacements for synthetic compounds.

Every year, the World Health Organization approximates that 6.8 million individuals succumb to neurological degenerative diseases, affecting more people across the world. This includes 45.9 million with epilepsy, 24 million with Alzheimer’s disease, and 35.6 million with dementia (Halliwell, 2001; Sayers, 2001; Siuly and Zhang, 2016; Cano et al., 2021). Approximately 600 different disorders influence the neurological system, including more prevalent conditions such as brain tumors, epilepsy, Parkinson’s disease, and stroke as well as dementia. These are characterized by confused state of mind with abnormal thinking and altered emotions, (Martinotti et al., 2021). Central, long, and fast acting inhibitors are applied to treat these memory impairment disorders (Birks and Harvey, 2018). Depression is another frequent and significant medical condition that negatively affects feelings and thinking and can reduce the capacity for routine work. These mental disorders also reduce quality of life, leading to moderate global health problems (Hasanat et al., 2019; Villas Boas et al., 2020). A class of pharmaceutical drugs known as antidepressants works by balancing neurotransmitter imbalances in the brain to reduce symptoms of depression and can also affect behavior and mental health (Lamers et al., 2011). Antidepressants like serotonin-norepinephrine reuptake inhibitors (SNRIs) and tricyclic antidepressants (TCAs) relieve depression and anxiety (Katzung, 2012). However, these drugs produce side effects in depressive patients. Therefore, the exploration of natural products as complementary and alternative approaches to mental health has attracted significant attention among researchers and healthcare professionals. This interest stems from the desire to broaden treatment options for mental health conditions, reduce side effects associated with conventional medications, and explore holistic approaches to well-being. Numerous studies and reviews highlight the potential benefits of natural products in promoting mental health and treating conditions such as anxiety and depression (Sarris et al., 2013).

Another perilous chronic metabolic illness is diabetes mellitus (DM), which is characterized by impaired glucose levels that disrupt the functioning of pancreatic beta cells. It is a modern epidemic that is spreading throughout the world; it is believed that by 2030 more than 439 million people will be affected by DM (Maqbool et al., 2019). It causes long-term effects on the heart, kidneys, liver, and eyes; the dysfunction and failure of various organs; and premature death (Salehi et al., 2019). Synthetic drugs such as acarbose and miglitol have potent inhibitory action against α-amylase and α-glucosidase enzymes to mitigate DM (Kaur et al., 2021). However, they produce more adverse effects such as abdominal flatulence, vomiting, and diarrhea (Sekhon-Loodu and Rupasinghe, 2019). These antidiabetic medications have made tremendous strides in the treatment of diabetes, but there are a number of drawbacks associated with their use, including toxicity, side effects, and drug resistance (Salehi et al., 2019). The adverse consequences of this chronic condition and its complications are amplified globally by the rising incidence of diabetes in young people. Conversely, approximately 80% of individuals diagnosed with diabetes rely significantly on medicinal plants as supplementary elements in their management protocols. This statistic emphasizes the substantial dependence of a considerable majority of diabetes patients on herbal remedies and plant-based supplements as complements to traditional medical treatments. The use of medicinal plants in this context might signify an increasing inclination toward complementary and alternative methods in diabetes care, with individuals actively seeking additional support from natural compounds alongside prescribed medications. This trend underscores the importance of adopting comprehensive healthcare strategies that seamlessly incorporate both conventional treatments and natural supplements to effectively address the various dimensions of diabetes management (Sekhon-Loodu and Rupasinghe, 2019; Yedjou et al., 2023).

*Byttneria pilosa* (Roxb.), commonly known as “Harijora,” belongs to the Malvaceae family. Its therapeutic attributes are well-acknowledged in the tribal cultures of Bangladesh and India (Ibrahim et al., 2015) and it is locally found in the forests of Chittagong, Chittagong Hill Tracts, Cox’s Bazar, and the Sylhet area. The crushed stems of *B. pilosa* (Roxb.) are traditionally applied topically to affected areas to treat syphilis, boils, rheumatism, and snake bites. In the Khumi community, a paste derived from the sensitive stem is utilized to mend damaged bones. In Tripura, elephantiasis is treated externally by applying the root of *B. pilosa* to afflicted areas (Jyoti et al., 2020). Phytochemical analysis of the whole plant has revealed the presence of phenols, tannins, steroids, alkaloids, glycosides, flavonoids, saponins, and terpenoids (Sikder et al., 2022). There has been limited exploration into the pharmacological potential of the leaf of *B. pilosa*. This study’s goal is to investigate aspects of this leaf’s medicinal properties. We specifically aimed to investigate the antioxidant, antidiabetic, anxiolytic, and antidepressant activities associated with the methanol extract derived from *B. pilosa* leaf (MEBP). Our comprehensive analysis sought to contribute valuable insights into the potential therapeutic benefits that *B. pilosa* leaf may offer in combating oxidative stress, managing diabetes, alleviating anxiety, and addressing depressive conditions.

## 2 Materials and methods

### 2.1 Plant collection and extraction

*B. pilosa* (Roxb.) specimens were gathered from Bhatiari, Chittagong, Bangladesh, based on a survey conducted among Chittagong communities regarding the utilization of plants for traditional treatments; the accession number for the collected specimens is 36186. Fresh leaves were naturally dried for 1 month and ground into a coarse powder. The ground leaf (310 mg) was soaked with 2 L 80% MeOH for 15 days with extemporaneous shaking. The liquid portion was separated through a filtration process, and then the filtrate was concentrated by rotary evaporation (Sterilin, United Kingdom). The concentrated gummy crude extract was stored at 4°C for further analysis.

### 2.2 Chemicals and reagents

Methanol, ascorbic acid, DPPH, gallic acid (Sigma Aldrich Co., St. Louis, MO, United States), Folin–Ciocalteu reagent (FCR) (Merck, India), and quercetin (Merck, India) were bought from local sources. Diazepam and imipramine were bought from Square Pharmaceuticals Limited and Aristopharma Limited, Bangladesh, respectively. All other chemicals were of analytical grade.

### 2.3 Assessment of antioxidant activity

#### 2.3.1 Free radical scavenging assay

The free radical scavenging activity of MEBP is based on the scavenging effect of the stable DPPH (Kumaran and Karunakaran, 2007). In brief, DPPH (w/v, 0.004%) 3.0 mL, MeOH solution was added to the various concentrations (500–15.625 μg/mL) of MEBP and ascorbic acid (standard). The mixture was incubated for 30 min at room temperature. Then, a UV-visible spectrophotometer was used to measure the absorbance at 517 nm against control. It was used to determine the percentage of inhibition [(A_0_–A_1_)/A_0_] × 100, where A_0_ denotes control absorbance and A_1_ denotes sample or standard absorbance.

#### 2.3.2 Reducing power capacity

Reducing the power capacity of the plant extract was based on the chemical changes of Fe (III) to Fe (II) transformation (Yen and Chen, 1995). In short, MEBP and ascorbic acid (500–15.626 μg/mL) 1.0 mL of solution was sequentially mixed with 2.5 mL of phosphate buffer (0.2 M, pH 6.6) and 2.5 mL of potassium ferricyanide (w/v, 1%). This mixture was incubated for 20 min at 50°C. Then, 2.5 mL of trichloroacetic acid (v/v, 10%) solution was added to the solution, and the mixture was passed through a centrifuge for 10 min at 3,000 rpm. Next, 2.5 mL of upper solution was collected using a pipette and transferred to a test tube, which was subsequently mixed with 2.5 mL of distilled water (DW) and 0.5 mL of ferric chloride solution (w/v, 0.1%). Finally, a UV-visible spectrophotometer was used to measure the absorbance at 700 nm.

### 2.4 Total phenolic content

The total phenolic content determination of plant extract was based on colorimetric oxidation/reduction reaction (Škerget et al., 2005). In brief, 2.5 mL of FCR solution (10 times diluted) was mixed with 0.5 mL of MEBP (concentration: 1 mg/mL) and GA (concentration: 500–15.625 μg/mL) solution. Then, 2.0 mL of sodium carbonate solution (w/v, 7.5%) was added to the mixture, which was subsequently incubated for 5 min at 50°C. Finally, a UV-visible spectrophotometer was used to measure the absorbance at 760 nm. The process was repeated thrice with the same concentration. To determine the phenolic content of MEBP, the GA calibration curve was used and revealed as mg of GA equivalent (GAE)/gm of the dried extract.

### 2.5 Total flavonoid content

A colorimetric assay is commonly used to determine the flavonoid content of plant extract (Oyedemi and Afolayan, 2011b; Oyedemi and Afolayan, 2011a). In short, 3 mL of MeOH, 0.2 mL of aluminum chloride (w/v, 10%), 0.2 mL of potassium acetate (1 M), and 5.6 mL of DW were added to 1.0 mL of MEBP (concentration: 1 mg/mL) and quercetin (concentration: 100–12.5 μg/mL) solution. Then, the mixture was incubated for 30 min at 25°C. Finally, a UV-visible spectrophotometer was used to measure the absorbance at 420 nm. The process was repeated thrice with the same concentration. To determine the flavonoid content of MEBP, the quercetin calibration curve was used and revealed as mg of quercetin equivalent (QE)/gm of the dried extract.

### 2.6 Antidiabetic activity by α-amylase

The assay was performed based on the starch–iodine test (Xiao et al., 2006). In brief, 1 mL of plant extract (concentrations: 1,000–125 μg/mL) was mixed with 1 mL of sodium phosphate buffer (pH 6.9, adjusted with NaCl) and 0.04 units of α-amylase solution. The mixture was then incubated at 37°C for 10 min. Next, 1 mL of starch solution (w/v, 1%) was added to the mixture solution, which was again incubated for 15 min at 37°C. To stop the enzymatic reaction, 40 μL of HCl solution (1 M) was added to the mixture as well as 200 μL of iodine solution. Finally, the mixture solution was subjected to a UV-visible spectrophotometer, and the absorbance was measured at 620 nm. The calculation of enzyme inhibition was determined thus:

$$\%\text{ Inhibition}=\left[\left(C–S\right)÷C\right]\times 100,$$

where S is the absorbance of the sample and C is the absorbance of the control.

### 2.7 Animals

Swiss albino mice (25–30 g) were selected and provided standard environment conditions, including food and water *ad libitum*. The animals underwent a 14-day acclimatization period and were additionally fasted overnight before conducting the experimental procedures. All the experiments were conducted in an isolated and noiseless state. All experimental protocols were approved by the IAEC (Institutional Animal Ethical Committee), Department of Pharmacy, International Islamic University Chittagong, Bangladesh (Pharm-P&D-61/08’16-130).

### 2.8 Assessment of acute toxicity

In accordance with ARRIVE guidelines, a randomized allocation process was employed to select mice, which were subsequently partitioned into five distinct groups comprising five mice each. Prior to experimentation, all mice underwent an overnight fasting period. Subsequently, oral administration of the MEBP extract was conducted, with doses ranging from 500 to 4,000 mg/kg, while the control group received only the vehicle (Percie du Sert et al., 2020)*.* Following the procedure, a meticulous observation period of 3–4 days was implemented to assess the mice closely for signs of allergic reactions such as swelling, skin rashes, and itching. Additionally, any instances of mortality were carefully documented during this monitoring period.

### 2.9 Assessment of anxiolytic activity

#### 2.9.1 Elevated plus-maze test

The EPMT was performed as per Rabbani et al. (2003). The equipment was constructed of two open (35 × 5 cm) and two closed (30 × 5 × 15 cm) arms that extended from a common central platform (5 × 5 cm), and the maze was situated at 60 cm height from the floor. The experimental procedure commenced by introducing mice onto the open arm of the EPM, with their orientation toward the center of the maze. Distinct groups of mice (*n* = 5) were subjected to different treatments: MEBP at varying doses (200 and 400 mg/kg, administered orally), diazepam (1 mg/kg, administered intraperitoneally), or a control solution (10 mL/kg, administered orally). These treatments were administered 30 min before the mice were placed on the EPM to ensure that the pharmacological effects had taken hold. The subsequent phase involved recording and analyzing the time spent by the mice in both the open and closed arms of the maze, providing insights into their exploratory and anxiety-related behaviors over a 5-min period.

#### 2.9.2 Light–dark box test

The LDBT was performed as per Jain et al. (2003). The equipment consisted of two boxes (25 × 25 × 25 cm) joined together. One box was made dark by roofing its top with plywood and the other box was illuminated with a light. In this experimental setup, a group of mice (*n* = 5) underwent specific treatments to explore the effects of MEBP (at doses of 200 and 400 mg/kg, administered orally) or a vehicle control (10 mL/kg, administered orally) given 60 min prior. Additionally, another group received diazepam (1 mg/kg, administered intraperitoneally) 30 min before being introduced into the light box. This approach facilitated a comprehensive investigation into the potential influence of MEBP and diazepam on the behavior of the mice in response to the environmental conditions within the light box. To begin the test session, a mouse was placed inside the light box positing to the central hole and the time spent in both boxes was recorded for 5 min.

### 2.10 Assessment of antidepressant activity

#### 2.10.1 Forced swimming test

The FST was conducted as per Nasrin et al. (2022). In this procedure, individual mice were placed in the FST apparatus, which consisted of an open cylindrical container with a diameter of 10 cm and a height of 25 cm. The container was filled with water 19 cm deep and maintained at 25°C ± 1°C. The mice were subjected to a 6-min testing period, during which the duration of immobility was measured. Immobility was defined as the point at which the mouse ceased active movement and remained floating motionless on the water. For the experimental interventions, groups of mice (*n* = 5) were administered treatments, including MEBP, at doses of 200 and 400 mg/kg (administered orally), imipramine (10 mg/kg, administered intraperitoneally), or a vehicle control (10 mL/kg, administered orally); finally, the immobility time of the mice was recorded.

#### 2.10.2 Tail suspension test

The TST was conducted as per Rodrigues et al. (2002). For the experimental treatments, groups of mice (*n* = 5) were administered with MEBP at doses of 200 and 400 mg/kg (administered orally), imipramine (10 mg/kg, administered intraperitoneally), or a vehicle control (10 mL/kg, administered orally). The administration of these substances took place 30 min prior to subjecting the mice to the TST apparatus. The subsequent phase involved recording the duration of immobility during the 6-min testing period.

### 2.11 Statistical analysis

Values were represented in mean ± SEM (standard error mean). *p < 0.05 and **p < 0.01 indicated statistical significance in comparison to the control group, followed by Dunnett’s test (GraphPad Prism v 7.0 and SPSS v 16).

### 2.12 Computational study

Molecular docking is a valuable computational tool, providing valuable insights into the pharmacological activities of natural products by elucidating the potential binding modes and affinities with target molecules. Two compounds (beta-sitosterol and sitosterol) were selected for computational analysis. The docking analysis was monitored as per Madhavi Sastry et al. (2013). Schrödinger Maestro (v11.1, 1540 Broadway, New York, United States) was used to optimize each phytochemical’s three-dimensional (3D) structure. The 3D structures of the following proteins were retrieved from the Protein Data Bank (https://www.rcsb.org/; Berman et al., 2000): urate oxidase (PDB: 1R4U) (Retailleau et al., 2004), glutathione reductase (PDB: 3GRS) (Karplus and Schulz, 1987), pancreatic alpha-amylase (PDB: 3BAJ) (Maurus et al., 2008), potassium channel (PDB ID: 4UUJ) (Lenaeus et al., 2014), human gamma-aminobutyric acid receptor (PDB ID: 4COF) (Miller and Aricescu, 2014), human serotonin transporter (PDB ID: 5I6X) (Coleman et al., 2016), and chimeric protein of 5-HT1B-BRIL (PDB ID: 4IAQ) (Wang et al., 2013).

### 2.13 ADME/toxicological property predictions

Lipinski’s rule of five was applied to each ligand. The drug likeliness and toxicological parameters of selected ligands were assessed using the QikProp tool (Schrödinger v11.1, 1540 Broadway, New York, United States) and AdmetSAR online prediction software, respectively (Lipinski et al., 2001; Daina et al., 2017).

## 3 Results

### 3.1 Effect of antioxidant activity by free radical scavenging assay

**FIGURE 1:**
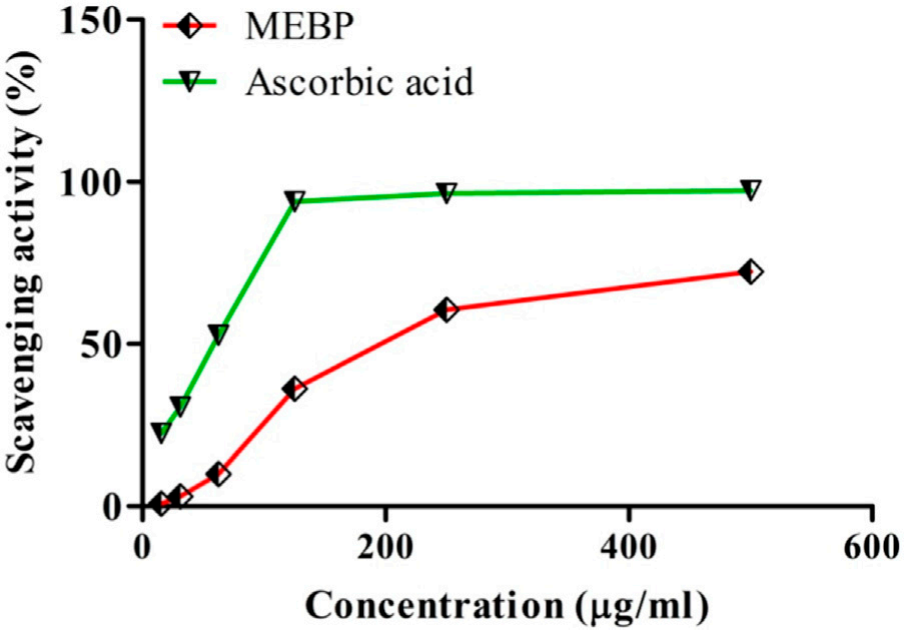
DPPH radical scavenging effect of MEBP and ascorbic acid.

The DPPH radical scavenging ability of MEBP and ascorbic acid is shown in Figure 1. From this, it is apparent that scavenging activity increases with concentration of MEBP and ascorbic acid increases, where the IC_50_ was found to be 290.56 and 55.31 μg/mL, respectively.

### 3.2 Effect of antioxidant activity by reducing power capacity

**FIGURE 2:**
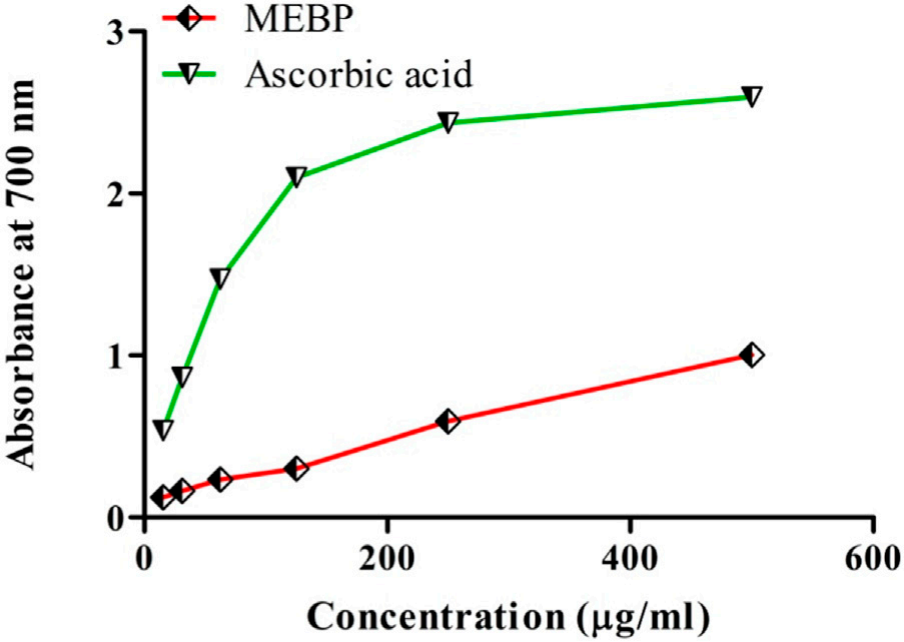
Reducing power of MEBP and ascorbic acid.

Reduction of the power activity of MEBP and ascorbic acid at various concentrations is shown in Figure 2. A concentration-dependent relationship was found, where MEBP comparatively increases its capacity like the ascorbic acid.

### 3.3 Effect of antioxidant activity by total phenol and flavonoid content

**FIGURE 3:**
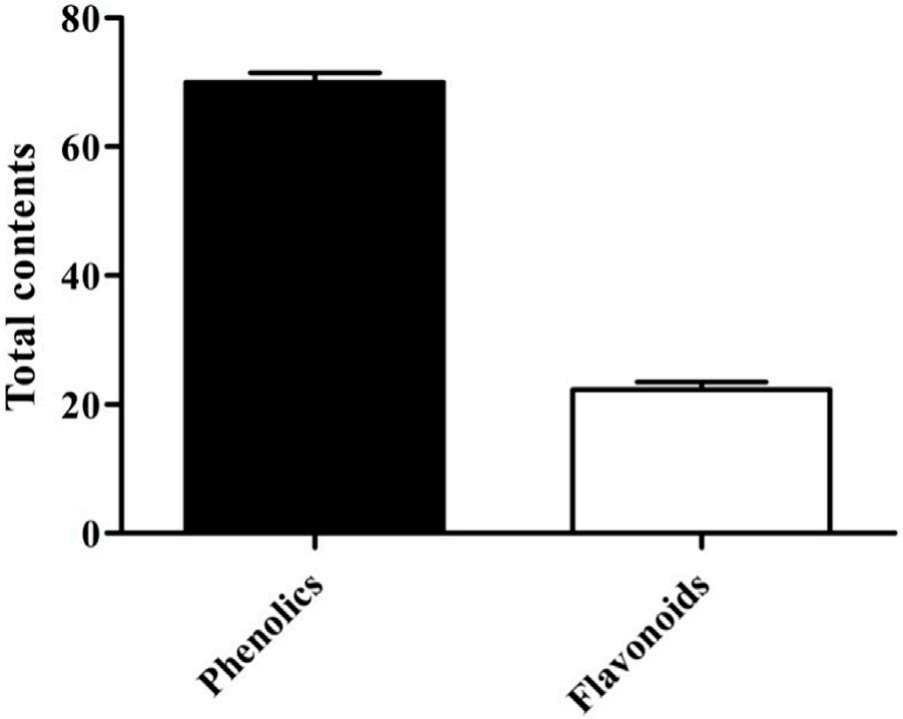
Total amount of phenolic and flavonoid contents of MEBP (*n* = 3).

The results of total phenol and flavonoid contents were calculated from the calibration curve of gallic acid and quercetin. The total phenolic and flavonoid contents were found to be 70 ± 1.53 (mg GAE/gm) and 22.33 ± 1.20 (mg QE/gm), respectively (Figure 3).

### 3.4 Effect of antidiabetic activity by α-amylase inhibition assay

**FIGURE 4:**
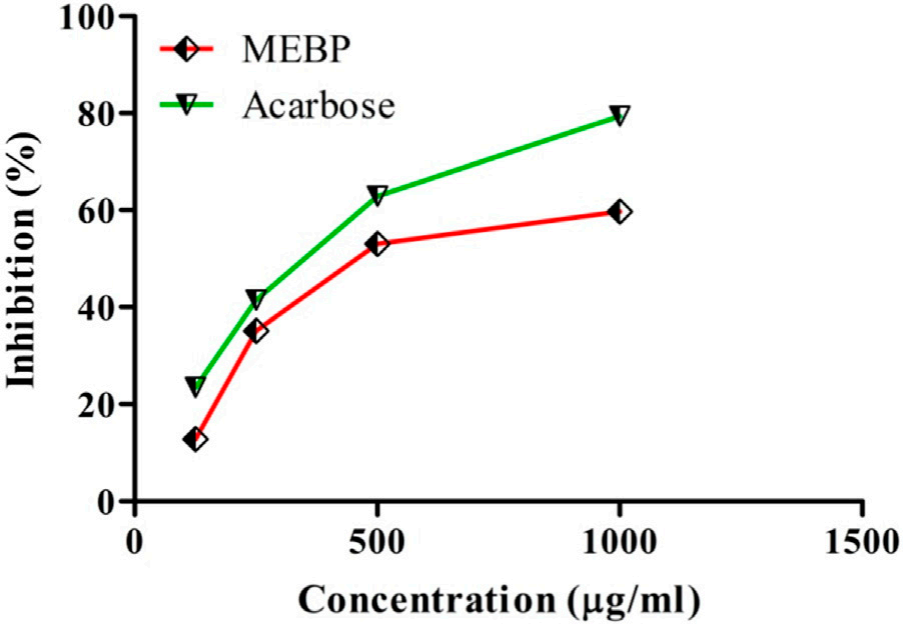
α-Amylase inhibitory effect of MEBP and acarbose.

The inhibitory effect of MEBP and acarbose on α-amylase is depicted in Figure 4. In comparison to acarbose, the percentage of α-amylase inhibition rose as the concentration of MEBP increased, reaching 50% inhibition at a concentration of 500 μg/mL.

### 3.5 Effect of acute toxicity

In this test, abnormal behaviors were not noticed at the given doses of MEBP (1,000, 2,000, 3,000, and 4,000 mg/kg). In addition, no mortality was found, which indicates that the median lethal dose of MEBP is higher than 4,000 mg/kg.

### 3.6 Neuro-modulatory effect of the elevated plus-maze test

**FIGURE 5:**
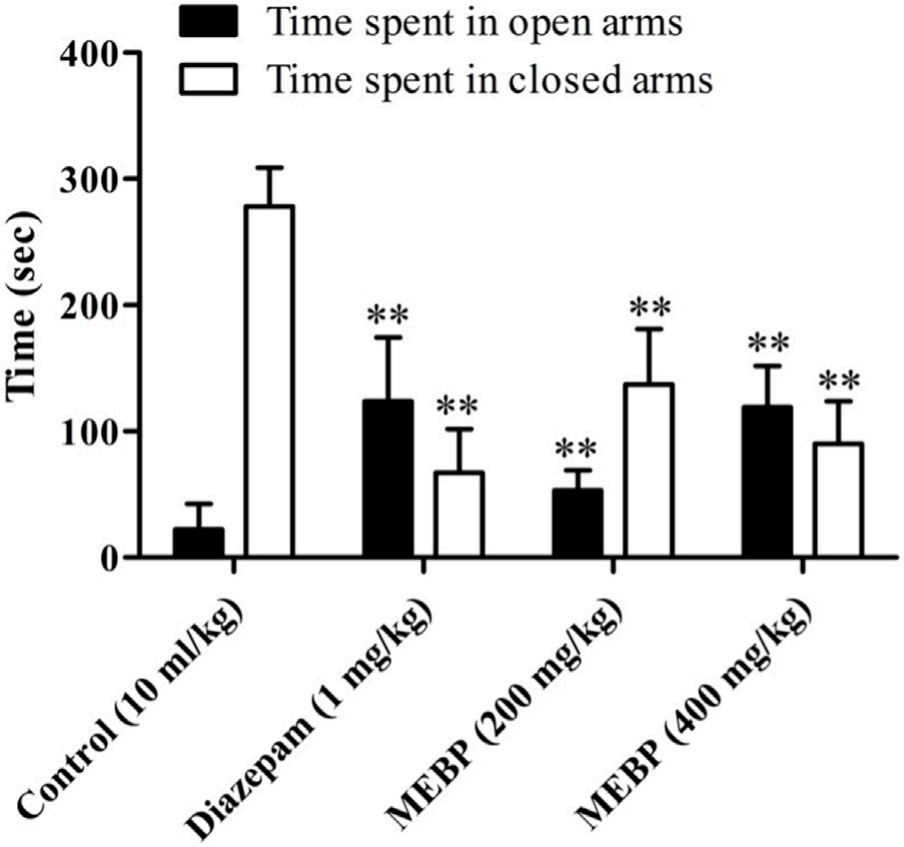
Effect of MEBP on the EPMT in mice (*n* = 5). Values are displayed as the mean ± SEM. **p < 0.01 compared with the control group (Dunnett’s test).

In the EPMT, animals treated with the vehicle spent 22.17 ± 20.19 s in the open arms and 278 ± 30.76 s in the closed arms. In contrast, the group treated with MEBP (200 and 400 mg) exhibited a dose-dependent response, leading to a significant increase and decrease in the time spent in the open and closed arms, respectively (200 mg: 53 ± 16.01 and 137 ± 44.11 s; 400 mg: 119 ± 32.87 and 90 ± 33.88 s; *p* < 0.01). Additionally, the reference standard diazepam (123.67 ± 50.67 and 67.33 ± 34.63 s; *p* < 0.01) demonstrated a similar behavior, and this observation was statistically significant (Figure 5).

### 3.7 Neuro-modulatory effect of the light–dark box test

**FIGURE 6:**
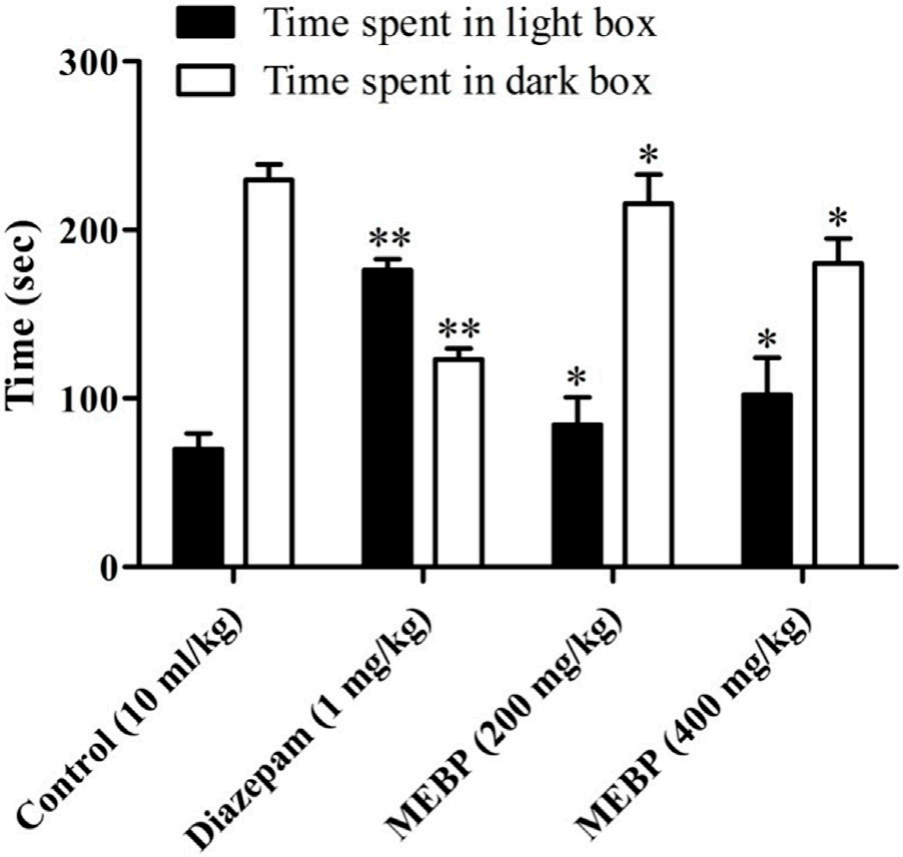
Effect of MEBP on the LDBT in mice (*n* = 5). Values are displayed as the mean ± SEM. *p < 0.05 and **p < 0.01 compared with the control group (Dunnett’s test).

In the LDBT, animals treated with vehicle spent 69.75 ± 9.43 s in the light box and 229.50 ± 9.34 s in the dark box (Figure 6). MEBP 200 mg (84.33 ± 16.22 and 215.67 ± 17.22 s; *p* < 0.05) and 400 mg (102 ± 22.07 and 180 ± 14.87 s; *p* < 0.05) markedly and dose-dependently increased with time spent in the light box and reduced with time in the dark box compared to the control group. Similarly, the reference standard diazepam (176 ± 6.62 and 123 ± 6.64 s; *p* < 0.01) also increased time spent in the light box and decreased time spent in the dark box.

### 3.8 Neuro-modulatory effect of the forced swimming test

**FIGURE 7:**
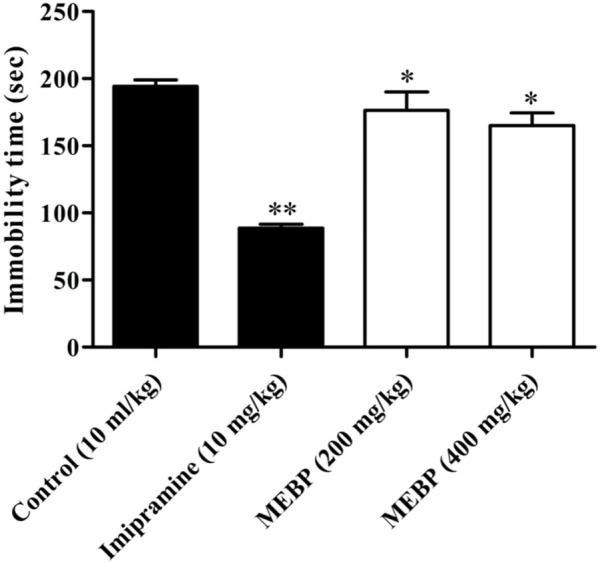
Effect of MEBP on the FST in mice (*n* = 5). Values are displayed as the mean ± SEM. *p < 0.05 and **p < 0.01 compared with the control group (Dunnett’s test).

In the FST, oral administration of MEBP at doses of 200 and 400 mg/kg resulted in a significant and dose-dependent reduction in the duration of immobility time (176.33 ± 13.68 and 165 ± 9.45 s; *p* < 0.05) compared to the control group (194.27 ± 4.81 s). The efficacy of MEBP in the FST was comparable to that of the antidepressant tricyclic imipramine at a 10 mg/kg dose (88.66 ± 2.93 s; *p* < 0.01) (Figure 7).

### 3.9 Neuro-modulatory effect of the tail suspension test

**FIGURE 8:**
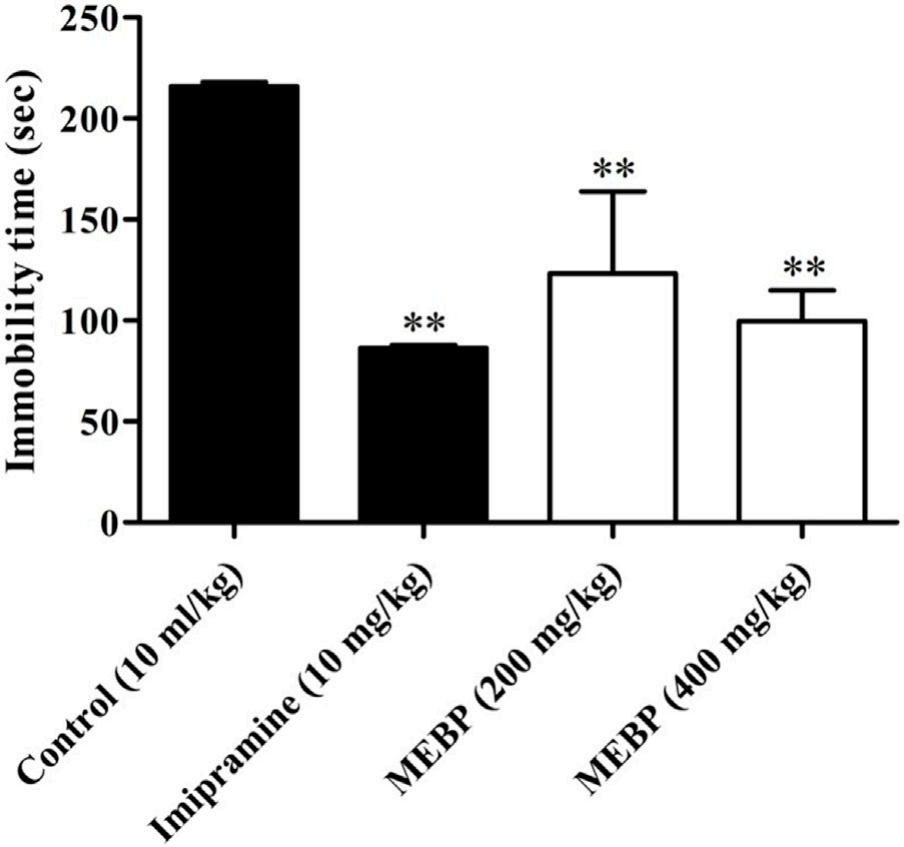
Effect of MEBP on the TST in mice (*n* = 5). Values are displayed as the mean ± SEM. **p < 0.01 compared with the control group (Dunnett’s test).

In the TST, MEBP (200 and 400 mg/kg) dose-dependently and significantly decreased the duration of immobility (123.33 ± 40.48 and 99.67 ± 15.30 s; *p* < 0.01) in comparison to the control group (217 ± 1.97 s). The efficacy of MEBP in the TST was comparable to that of the tricyclic antidepressant imipramine 10 mg/kg (86.4 ± 1.35 s; *p* < 0.01) (Figure 8).

### 3.10 Effect of *in silico* study

**FIGURE 9:**
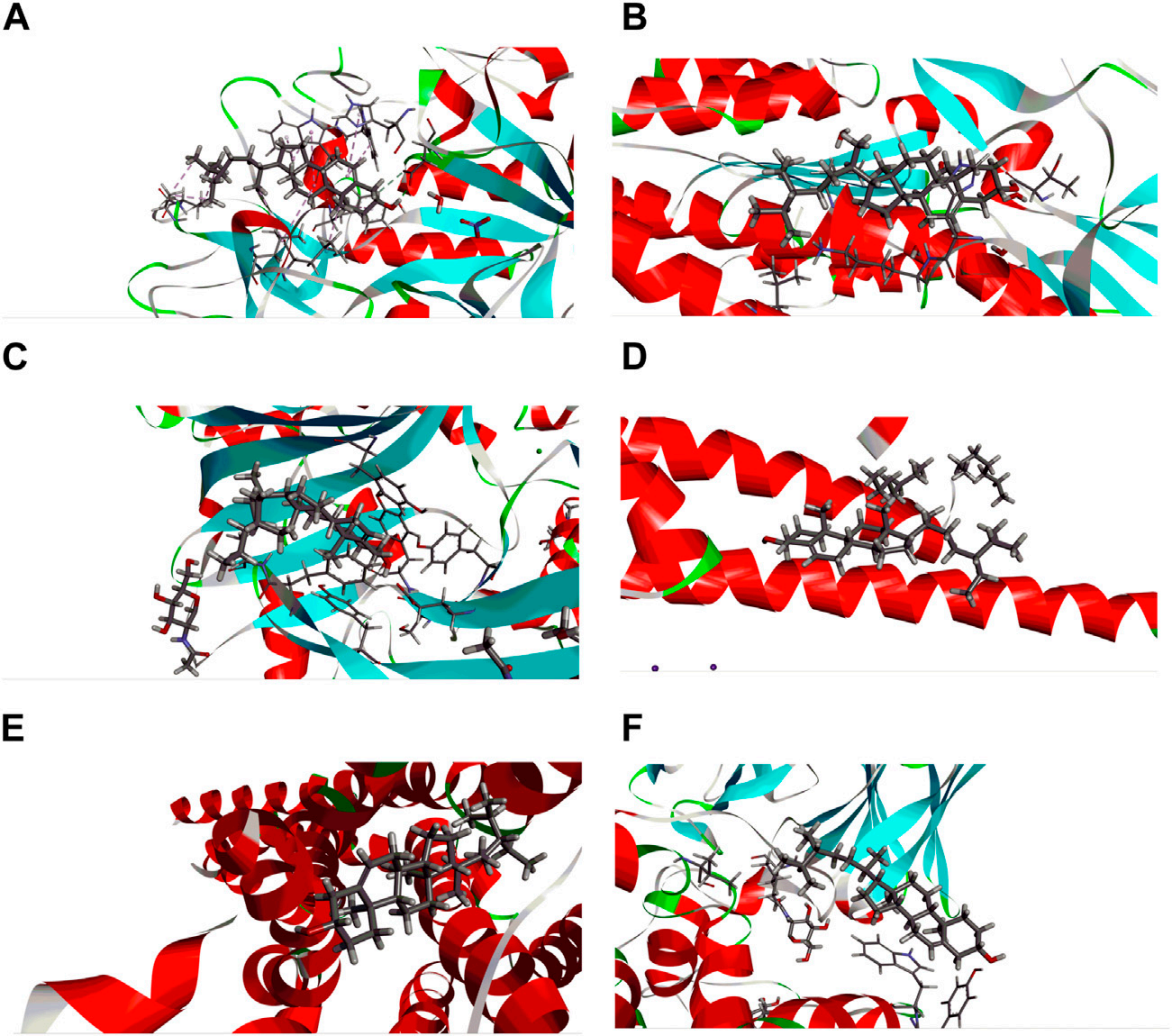
Best ranked docking pose and interactions between beta-sitosterol and sitosterol with **(A)** human pancreatic alpha-amylase (PDB ID: 3BAJ), **(B)** glutathione reductase (PDB ID: 3GRS), **(C)** human gamma-aminobutyric acid receptor (PDB ID: 4COF), **(D)** potassium channel (PDB ID: 4UUJ), **(E)** chimeric protein of 5-HT1B-BRIL (PDB ID: 4IAQ), and **(F)** human serotonin transporter (PDB ID: 5I6X).

**FIGURE 10:**
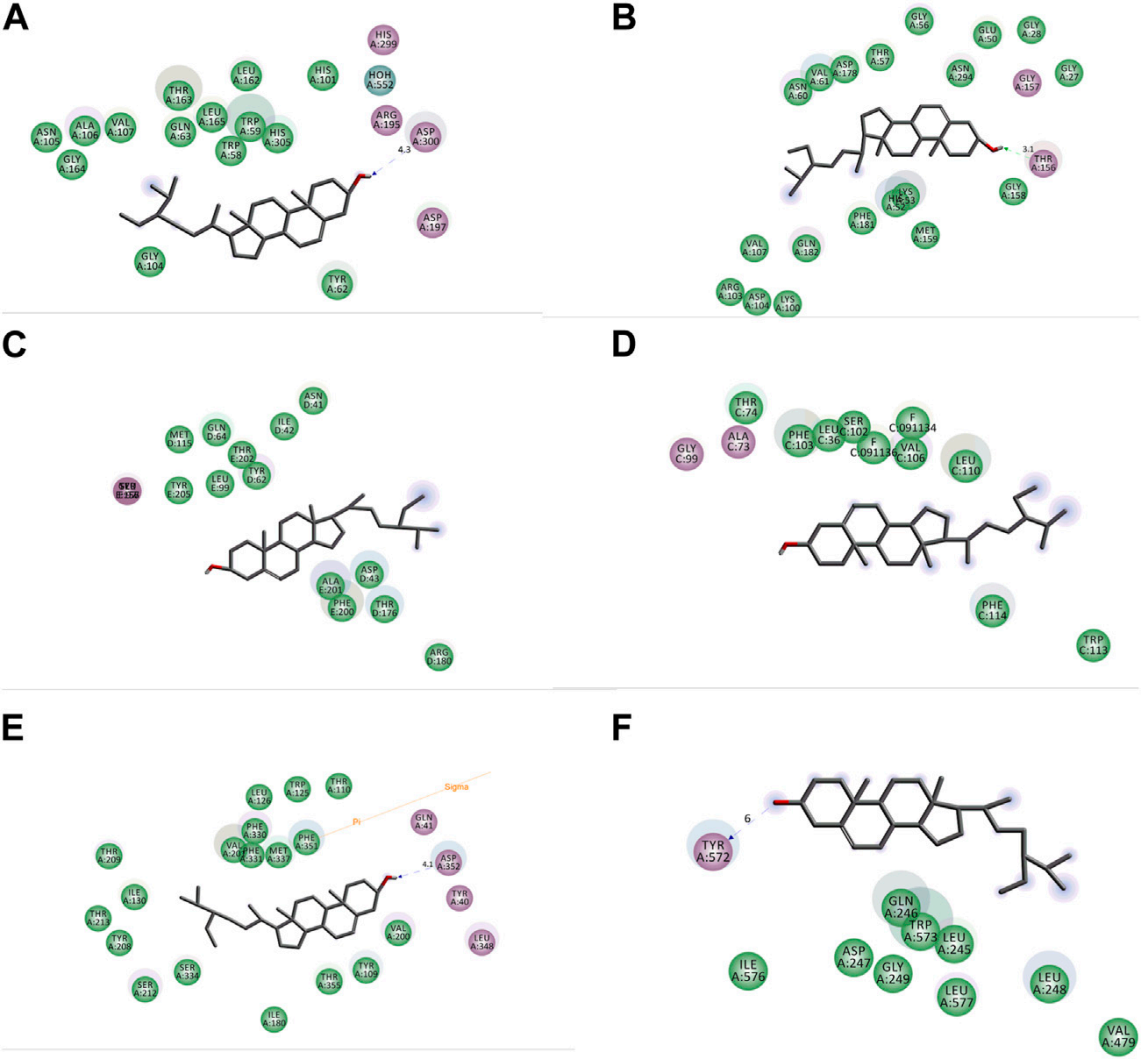
2D representation of interactions between beta-sitosterol and sitosterol with **(A)** human pancreatic alpha-amylase (PDB ID: 3BAJ), **(B)** glutathione reductase (PDB ID: 3GRS), **(C)** human gamma-aminobutyric acid receptor (PDB ID: 4COF), **(D)** potassium channel (PDB ID: 4UUJ), **(E)** chimeric protein of 5-HT1B-BRIL (PDB ID: 4IAQ), and **(F)** human serotonin transporter (PDB ID: 5I6X).

In this study, pharmacological activity of natural products (beta-sitosterol and sitosterol) molecular docking was performed. Beta-sitosterol and sitosterol were specifically chosen as subjects for the docking analysis to gain a more comprehensive understanding of the underlying mechanisms that drive the noteworthy biological activities observed in MEBP. The docking score results are shown in Table 1 and Figures 9, 10. Isolated selected compounds of beta-sitosterol and sitosterol exhibited −3.412, −3.312, and −4.351 kcal/mol docking scores against urate oxidase (PDB ID: 1R4U) and glutathione reductase (PDB ID: 3GRS), respectively, for antioxidant activity, although the compound showed no binding affinity with the 1R4U protein. For antidiabetic activity docking analysis, beta-sitosterol and sitosterol showed −5.312 and −5.213 kcal/mol against human pancreatic alpha-amylase (PDB ID: 3BAJ). In addition, this study showed that beta-sitosterol and sitosterol displayed −3.539 and −3.552 kcal/mol docking scores against the human gamma-aminobutyric acid receptor (PDB ID: 4COF) and displayed −2.963 and −2.975 kcal/mol docking scores against the potassium channel (PDB ID: 4UUJ) for anxiolytic activity. Furthermore, they displayed the highest docking score of −5.859 and −6.875 kcal/mol against the chimeric protein of 5-HT1B-BRIL (PDB ID: 4IAQ) while exhibiting −3.136 and −4.132 kcal/mol docking scores against the human serotonin transporter (PDB ID: 5I6X) for antidepressant activity.

**TABLE 1:** Docking scores of selected phytochemicals from MEBP against 1R4U, 3GRS K+ channel (4UUJ), SERT3 (PDB ID: 5I6X), and 3BAJ for antioxidant, anxiolytic, antidepressant, and antidiabetic activities, respectively.

Selected compound	1R4U	3GRS	3BAJ	4COF	4UUJ	5I6X	4IAQ
Beta-Sitosterol	−3.412	−4.351	−5.312	−3.539	−2.963	−3.136	−5.895
Sitosterol	−3.312	−4.351	−4.312	−3.552	−2.975	−4.132	−6.875

1R4U and 3GRS PDB ID for antioxidant activity; 3BAJ PDB ID for antidiabetic activity; 4COF and 4UUJ PDB ID for anxiolytic activity; 5I6X and 4IAQ PDB ID for antidepressant activity.

## 4 Discussion

The research was crafted with the primary objective of examining the multifaceted therapeutic effects of MEBP by focusing on its antioxidant, anxiolytic, antidepressant, and antidiabetic properties. Within the framework of this investigation, MEBP exhibited noteworthy antioxidant capabilities, demonstrating a concentration-dependent efficacy in scavenging free radicals. These are known for their propensity to attack vital macromolecules and pose a substantial threat by causing cellular damage and disrupting homeostasis. The repercussions of these deleterious free radical reactions extend to being implicated as the underlying cause in a myriad of degenerative diseases, prominently featuring oxidative stress, cancer, atherosclerosis, diabetes, asthma, and the aging process (Chaudhary et al., 2023). Accumulating evidence underscores the pivotal role of these harmful reactions in the onset and progression of a spectrum of health conditions, thereby emphasizing the critical importance of antioxidants, such as those present in MEBP, in mitigating the adverse effects of oxidative stress and potentially influencing disease outcomes. The responsibility for attenuating oxidative stress or combating cancer may be attributed to the presence of beta-sitosterol and other phytoconstituents within MEBP.

Amylase, a key enzyme, operates across multiple pathways, including the reduction of postprandial glucose levels, the delay in metabolism and absorption, and the breakdown of starch. The multifaceted actions of amylase in these pathways suggest potential benefits, with implications for mitigating insulin resistance (Miura et al., 2001). Long-standing diabetes mellitus is linked to a spectrum of complications, including but not limited to atherosclerosis, myocardial infarction, neuropathy, and nephropathy. The association between these complications and prolonged diabetes has traditionally been attributed to the persistent elevation of glucose levels, leading to subsequent oxidative stress. The mechanisms contributing to heightened oxidative stress in diabetes involve non-enzymatic glycosylation, auto-oxidative glycosylation, and metabolic stress. Non-enzymatic glycosylation refers to the process by which glucose molecules react with proteins in a non-enzymatic manner, leading to the formation of advanced glycation end products (AGEs). Auto-oxidative glycosylation involves the oxidation of glucose without the involvement of enzymes, generating reactive oxygen species (ROS) and contributing to oxidative stress. Additionally, metabolic stress, arising from dysregulated glucose metabolism, further exacerbates oxidative stress in individuals with diabetes (Nishikawa et al., 2000; Brownlee, 2001; Rains and Jain, 2011). This investigation sought to elucidate and expand our understanding of the antidiabetic properties of the tested compounds, shedding light on their potential therapeutic implications in managing glucose metabolism and insulin resistance. It was observed that the inhibition of α-amylase exhibited a noteworthy and dose-dependent response upon the administration of MEBP. The inhibitory effect demonstrated a significant correlation with the dosage of MEBP, indicating a progressive and dose-responsive reduction in α-amylase activity. This inhibition also indicates the presence of some phytoconstituents such as polyphenols, flavonoids, saponins, steroids, and terpenoids.

In addition to investigating its anxiolytic and antidepressant activity, this study considered the potential anxiolytic and antidepressant properties of MEBP with the aim of evaluating its efficacy as a candidate for anxiolytic and antidepressant-like agents. A diverse range of animal behavioral models was employed to assess these effects, and various doses of MEBP were administered accordingly. Among the diverse array of models considered, the EPMT and LDBT emerged as particularly prominent and widely utilized for discerning anxiolytic effects in rodents. Notably, the EPMT and LDBT are established as primary models for investigating the modulation of γ-aminobutyric acid receptors, which are pivotal in the context of anxiolytic drug assessment. By employing these sophisticated behavioral paradigms, this study sought to provide a nuanced understanding of the potential of MEBP as an agent with anxiolytic and antidepressant-like properties, contributing valuable insights to the field of psychopharmacology. The anxiolytic efficacy of EPMT and LDBT was indicated by increasing time spent in the open arm or light box and decreasing time spent in the closed arm or dark box (Rishikesh et al., 2012). In the present investigation, MEBP showed a dose-dependent and significant time-spent in EPMT, where MEBP increased time spent in the open arm and decreased time spent in the closed arm. In the LDBT, MEBP displayed similar behavior for EPMT where MEBP increased time spent in the light box and decreased time spent in the dark box. Furthermore, the present result suggests that MEBP exhibited anxiolytic-like activity in both models. However, the antidepressant activity of MEBP was screened by the two most common behavioral models: FST and TST. Decreasing immobility time in FST and TST reflects antidepressant activity (Santosh et al., 2011; Fahad et al., 2021). Both these models are quite sensitive to various established antidepressant drugs like serotonin-specific reuptake inhibitors, tricyclics, and monoamine oxidase inhibitors, and it is also reported that FST and TST induce conditions similar to human depression (Hillhouse and Porter, 2015). This aligns with existing literature on natural products and their psychopharmacological implications. Alkaloids and polyphenols, with their CNS modulating effects, have demonstrated potential in influencing anxiolytic and antidepressant responses (Guha et al., 2021; Tayab et al., 2022).

As a well-established structural drug design technique, molecular docking has been widely used to model interactions between drug molecules and target proteins at the atomic scale. This makes it possible to interpret the initial biochemical mechanisms and outline the properties of the molecule at the target protein’s active site (Joca et al., 2019). Docking aims to accurately anticipate the ligand’s orientation within the target protein’s binding pocket and obtain an approximation of the binding strength from the docking score. This enables us to understand the links between their structure and activities and to put molecules derived from nature into context (Tayab et al., 2021). The docking experiment, targeted at unraveling the fundamental potential antioxidant, antidiabetic, anxiolytic, and antidepressant activity of *B*. *pilosa* (Roxb.) leaf, focused on seven target proteins: urate oxidase, glutathione reductase, human pancreatic alpha-amylase, human gamma-aminobutyric acid receptor, potassium channel, 5-HT1B-BRIL, and human serotonin transporter. The enzymatic conversion of uric acid is facilitated by urate oxidase, leading to the formation of 5-hydroxyisourate (Motojima et al., 1988). Simultaneously, the maintenance of an adequate supply of reduced glutathione is orchestrated by the action of glutathione reductase. The pivotal role played by glutathione in ensuring proper cellular function is underscored, serving as a crucial element in both sustaining cellular health and averting oxidative stress within human cells (Deponte, 2013). Urate oxidase from *Aspergillus flavus* (PDB ID: 1R4U) and glutathione reductase (PDB ID: 3GRS) both showed good binding energies against beta-sitosterol. A mechanistic study of the essential allosteric activation of human pancreatic alpha-amylase (PDB ID: 3BAJ) where a chloride ion has been directed by exploring a wide range of anion substitutions through kinetic and structural experiments (Maurus et al., 2008). Human pancreatic alpha-amylase (PDB ID: 3BAJ) possesses significant binding affinity with beta-sitosterol. Type-A γ-aminobutyric acid (GABA) receptors are pivotal in promptly inhibiting synaptic transmission within the human brain, serving as the primary mediators (Miller and Aricescu, 2014). Conversely, potassium channels exert significant influence by regulating the membrane potential across various cell types. These channels facilitate the passive movement of potassium ions through the cell membrane, contributing to the intricate orchestration of cellular processes (Lenaeus et al., 2014). From both potassium channels (PDB ID: 4UUJ) and human gamma-aminobutyric acid receptors (PDB ID: 4COF), human gamma-aminobutyric acid receptor presented better binding affinity against beta-sitosterol. Through the sodium- and chloride-dependent reuptake of neurotransmitters into presynaptic neurons, the serotonin transporter (SERT) terminates serotonergic signaling. By blocking reuptake and prolonging neurotransmitter signaling, antidepressant and psycho-stimulant drugs targeted SERT. SERT drops the levels of 5-HT at the SC by transferring them to the presynaptic cells where they are either repackaged into secretory vesicles or metabolized by MAO-A. These findings underscore the potential therapeutic implications of beta-sitosterol in the development of antidepressant treatments. The observed interactions provide valuable insights that contribute to ongoing efforts in designing novel and effective strategies for managing depressive disorders.

## 5 Conclusion

The present study’s outcomes shed light on the moderate antioxidant and antidiabetic activities exhibited by MEBP. Additionally, noteworthy effects were observed in anxiolytic and antidepressant models. Despite these promising findings, further research is needed to comprehensively elucidate the underlying mechanism of MEBP and ascertain its full therapeutic potential.

## Data Availability

The original contributions presented in the study are included in the article/Supplementary material; further inquiries can be directed to the corresponding authors.
